# CK1δ in lymphoma: gene expression and mutation analyses and validation of CK1δ kinase activity for therapeutic application

**DOI:** 10.3389/fcell.2015.00009

**Published:** 2015-02-20

**Authors:** B. Sophia Winkler, Franziska Oltmer, Julia Richter, Joachim Bischof, Pengfei Xu, Timo Burster, Frank Leithäuser, Uwe Knippschild

**Affiliations:** ^1^Department of Pathology, Ulm University HospitalUlm, Germany; ^2^Department of General and Visceral Surgery, Surgery Center, Ulm University HospitalUlm, Germany; ^3^Department of Neurosurgery, Ulm University HospitalUlm, Germany

**Keywords:** CK1, lymphoma, therapy, inhibitor, mutation analysis, cell cycle

## Abstract

The prognosis of lymphoid neoplasms has improved considerably during the last decades. However, treatment response for some lymphoid neoplasms is still poor, indicating the need for new therapeutic approaches. One promising new strategy is the inhibition of kinases regulating key signal transduction pathways, which are of central importance in tumorigenesis. Kinases of the CK1 family may represent an attractive drug target since CK1 expression and/or activity are associated with the pathogenesis of malignant diseases. Over the last years efforts were taken to develop highly potent and selective CK1-specific inhibitor compounds and their therapeutic potential has now to be proved in pre-clinical trials. Therefore, we analyzed expression and mutational status of CK1δ in several cell lines representing established lymphoma entities, and also measured the mRNA expression level in primary lymphoma tissue as well as in non-neoplastic blood cells. For a selection of lymphoma cell lines we furthermore determined CK1δ kinase activity and demonstrated therapeutic potential of CK1-specific inhibitors as a putative therapeutic option in the treatment of lymphoid neoplasms.

## Introduction

Lymphoid neoplasms are heterogeneous malignancies of the hematopoietic and lymphoid tissues (Jaffe et al., 2001; Harris et al., 2008). In the United States, the annual incidence of all lymphoid malignancies is about 34 per 100,000.

According to the current WHO classification lymphoid neoplasms are divided into Hodgkin-(HL) and Non-Hodgkin-Lymphomas (NHL) (Jaffe et al., 2001; Harris et al., 2008). HL represents approximately 30% of lymphoid neoplasms and is dichotomized into classical Hodgkin Lymphoma and nodular lymphocyte predominant Hodgkin lymphoma. Although Hodgkin lymphoma is of B cell origin with only very few exceptions, its distinct histology, immunophenotype, and clinical presentation justify separating it from other mature B cell neoplasms (Jaffe et al., 2001; Schmitz et al., 2009). Mature NHL are broadly divided into B and T cell neoplasms and further sub-classified into a large number of distinct entities, according to phenotype, genotype and clinical properties. One of the major entities of mature B cell neoplasms is diffuse large B cell lymphoma, not otherwise specified (NOS), that contains various subtypes and morphologic variants, including immunoblastic lymphoma (Menon et al., 2012). Other B cell lymphoma entities are primary mediastinal B cell lymphoma and Burkitt lymphoma. Primary mediastinal B cell lymphoma is a diffuse large B cell lymphoma typically arising in the mediastinum (Stein et al., 2008). Burkitt lymphoma constitutes an aggressive, highly proliferative mature B cell lymphoma often located at extranodal sites (Leoncini et al., 2008). B and T lymphoblastic lymphomas are primarily pediatric, immature tumors belonging to the group of precursor lymphoid neoplasms (Borowitz and Chan, 2008).

Owing to increasingly elaborate therapy protocols, the prognosis of lymphoid neoplasms has continuously improved during the last decades. Nevertheless, for a significant number of lymphoid neoplasms the response to established treatment remains unsatisfactory, highlighting the need for new therapy strategies. Currently, one of the most promising approaches in oncology seems to be targeting of growth-promoting protein kinases by specific inhibitory substances. Such kinases have already been identified for a few lymphoid neoplasms, i.e., chronic lymphocytic leukemia, mantle cell lymphoma (Aalipour and Advani, 2014) and hairy cell leukemia (Dietrich et al., 2012). However, for the vast majority of malignant lymphomas suitable target molecules are still unknown. Therefore, large research interest is now being focused on the identification of new specific protein kinases to interrupt pivotal signaling pathways in lymphoma cells.

Casein kinase 1 (CK1) isoforms are ubiquitously expressed serine/threonine-specific kinases. In human, at least six different isoforms (α, γ1, γ2, γ3, δ, and ε) and a number of related splice variants have been identified (Green and Bennett, 1998; Fu et al., 2001; Burzio et al., 2002; Knippschild et al., 2014). While all isoforms and variants significantly differ in length and composition of their N- and C-terminal domains, the kinase domain is highly conserved within all isoforms. CK1δ and ε show the highest sequence homology of about 98%. CK1 family members are able to phosphorylate a broad range of substrates regulating important cellular processes like cytokinesis, DNA repair, cell cycle progression, differentiation processes, and apoptosis (Knippschild et al., 2005, 2014; Price, 2006; Cheong and Virshup, 2011). CK1 activity is also linked to pathways involving the central signal transduction molecules p53 or β-catenin (Cheong and Virshup, 2011; Cruciat, 2014; Knippschild et al., 2014). Accordingly, changes in cellular CK1 expression and/or activity contribute to the pathogenesis of various diseases including cancer. In recent years considerable research effort has addressed the development and characterization of CK1-specific inhibitors (for review see Knippschild et al., 2014). As a result, new inhibitor compounds specifically targeting CK1 isoforms are more and more proving their therapeutic potential for a variety of malignancies (Perez et al., 2010; Knippschild et al., 2014).

To investigate whether CK1δ could be a potential therapeutic target in lymphoid malignancies, we characterized the role of CK1δ in 18 human cell lines representing a number of established lymphoma entities, i.e., acute lymphoblastic B cell lymphoma, acute lymphoblastic T cell lymphoma, diffuse-large B cell lymphoma (NOS), primary mediastinal B cell lymphoma, Burkitt lymphoma, classical Hodgkin-lymphoma, and nodular lymphocyte-predominant Hodgkin Lymphoma. In the present study, we tested the effects of established and novel CK1-specific inhibitor compounds on selected lymphoma cell lines in order to evaluate the therapeutic potential of CK1 inhibition. Prior to functional testing, we determined expression, mutational status, and kinase activity of CK1δ in several established lymphoma cell lines to estimate the effects of CK1-specific kinase inhibition and to correlate kinase inhibition-mediated effects with CK1δ expression and/or activity levels or possible mutations in the CK1δ coding sequence.

## Materials and methods

### Human lymphoma tissue

In summary 12 patients suffering from diffuse large B cell lymphomas whose informed consent was obtained prior to surgery were included in the study. Lymphoma diagnosis was in accordance with the current World Health Organization classification. All samples were drawn from our archive of frozen tissues and pseudonymized to comply with the German law for correct usage of archival tissue for clinical research (Deutsches Ärzteblatt 2003; 100 A1632). Frozen tissue samples were used for gene expression analysis. The project was performed with the permission of the independent local ethics committee of the University of Ulm.

### Cell lines

Within the present study the following established lymphoma cell lines were used: DAUDI (Klein et al., 1968), DEV (Poppema et al., 1985), DOHH-2 (Kluin-Nelemans et al., 1991), HDLM2 (Drexler et al., 1986), JIYOYE (Pulvertaft, 1965), Jurkat (Schneider et al., 1977), KARPAS-1106P (Nacheva et al., 1994), KM-H2 (Kamesaki et al., 1986), L-1236 (Kanzler et al., 1996), L-428 (Diehl et al., 1981), L-540 (Diehl et al., 1981), MedB-1 (Moller et al., 2001), NALM-6 (Hurwitz et al., 1979), RAJI (Pulvertaft, 1964), RAMOS (Klein et al., 1975), SU-DHL-6 (Epstein et al., 1978), SU-DHL-8 (Epstein et al., 1978), U-H01 (Mader et al., 2007). Furthermore, the Epstein-Barr virus (EBV) transformed lymphoblastoid B cells from peripheral blood of healthy donors LCL1, LCL2, and BSM (Halder et al., 2000) were used. All cell lines were maintained in RPMI-1640 medium (Invitrogen, Karlsruhe, Germany) except for U-H01 (4:1 mixture of DMEM and RPMI-1640; Invitrogen, Karlsruhe, Germany) and MedB-1 (IMDM; Invitrogen, Karlsruhe, Germany). Media were supplemented with 2 mM glutamine, 100 units/ml penicillin, 100 μg/ml streptomycin and 10% heat-inactivated fetal calf serum (FCS) except for DEV, HDLM2, Jurkat, L-540, for which 20% FCS was used. Cells were kept in a humidified 5% carbon dioxide atmosphere at 37°C.

### Gene expression analysis

**Figure 1:**
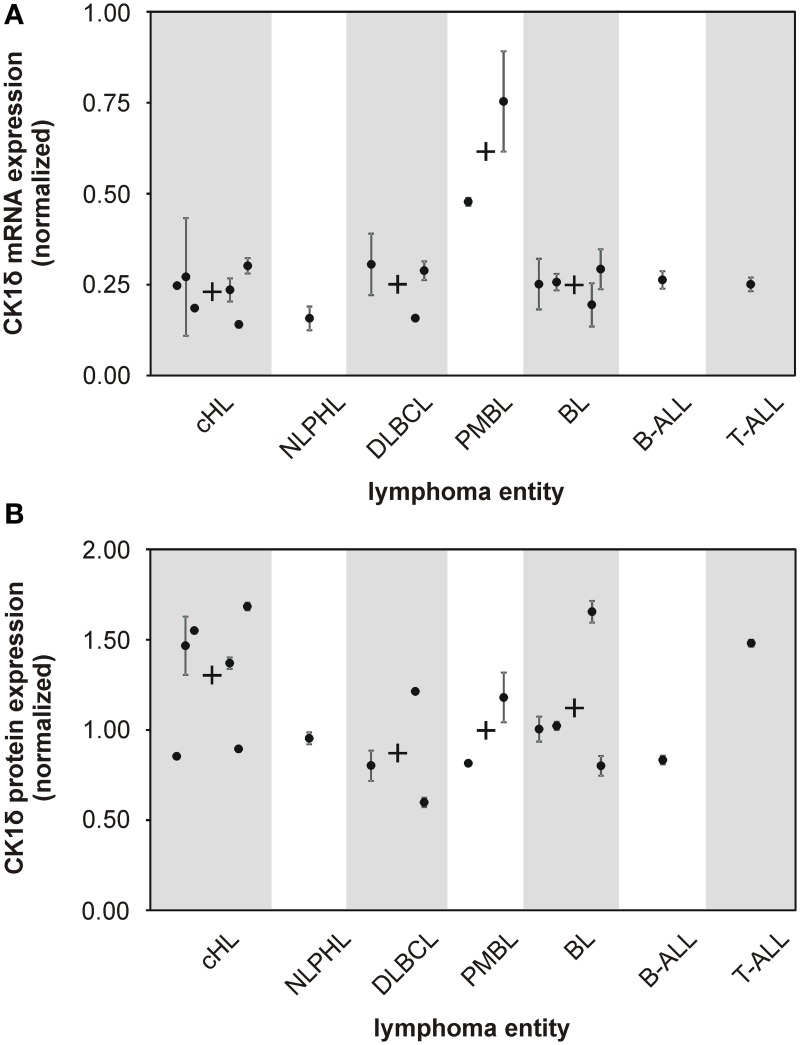
**mRNA and protein expression levels of CK1δ in various established malignant lymphoma cell lines**. The expression levels of CK1δ in various established malignant lymphoma cell lines were analyzed by quantitative RT-PCR **(A)** and quantitative Western blot analyses **(B)**. Black dots represent gene or protein expression of one cell line relative to tonsil reference material. For clear presentation cell lines were assigned to their entity of lymphoma. Each black dot of gene expression analysis is representative for three replicates. Error bars indicate the fold-change range for the calculated ΔΔCt values. For quantitative Western blot analyses data from one representative analysis out of three are shown. In case more than one cell line per lymphoma entity was tested, mean values are indicated by a black cross. Abbreviations: *CK1δ*: Casein Kinase 1 isoform delta, c*HL*: classical Hodgkin lymphoma including HDLM2, KM-H2, L-1236, L-428, L-540, and U-H01 cells, *NLPHL:* nodular lymphocyte predominant Hodgkin lymphoma represented by DEV cells, *DLBCL*: diffuse large B cell lymphoma containing DOHH-2, SU-DHL-6, and SU-DHL-8 cells, *PMBL:* primary mediastinal large B cell lymphoma including KARPAS-1106P and MedB-1 cells, *BL:* Burkitt lymphoma including DAUDI, JIYOYE, RAJI, and RAMOS cells, B-*ALL:* B cell acute lymphoblastic lymphoma represented by NALM-6 cells, *T-ALL*: T cell acute lymphoblastic lymphoma represented by JURKAT cells.

Total RNA was isolated from human lymphoma cells, blood cells, and tumors from lymphoid tissue using the RNeasy Mini kit (Qiagen, Hilden, Germany). Equal amounts of total RNA were reverse transcribed into complementary DNA using the AffinityScript cDNA Synthesis kit (Agilent Technologies, Waldbronn, Germany). Quantitative reverse-transcription PCR (qRT-PCR) data presented in Figure 1 were generated using an iCycler real-time PCR system and iQ SYBR green supermix according to the manufacturer's instructions (both Biorad, Munich, Germany). Glycerinaldehyd-3-phosphat-dehydrogenase (GAPDH) was used as external standard, cryopreserved tissue of human tonsils as reference material. qPCR-conditions were denaturation at 95°C, annealing at 60°C (CK1δ-specific primers), and elongation at 72°C in 40 cycles. CK1δ was amplified using oligonucleotide primers AGCACATCCCCTATCGTGAG and CGTAGCCCAGAGACTCCAAG. SYBR green fluorescent signals were detected at the end of each annealing step. Relative gene expression of CK1δ in lymphoma cells was calculated using the ΔΔCt method. Real-time RT-PCR data shown in **Figure 7** were generated using the QuantiTect™ SYBR Green PCR kit (Qiagen, Hilden, Germany) and a LightCycler® 480 (Roche, Mannheim, Germany) real-time PCR instrument. QuantiTect Primer Assays for CK1δ and HPRT were purchased from Qiagen (Qiagen, Hilden, Germany) and used according to the manufacturer's instructions using the following cycling conditions: heat activation at 95°C for 15 min, 55 cycles of 15 s denaturation at 94°C, 20 s annealing at 55°C and 30 s synthesis at 72°C. Slope was always set at 20°C/s throughout amplification. The fluorescence data collection of the color signal was measured in real-time model at the end of each DNA synthesis cycle, and the crossing point was calculated. The specificity of DNA amlplification for CK1δ was examined analyzing the melting curve. mRNA contents were normalized to HPRT mRNA levels.

### Isolation of primary human immune cells

Human peripheral blood mononuclear cells (PBMCs) were freshly isolated from buffy coats of healthy blood donors by density gradient centrifugation. Conventional dendritic cells (cDC, CD1c^+^), monocytes (CD14^+^), T regulatory cells (CD4^+^, CD25^+^), cytotoxic T lymphocytes (CD8^+^), natural killer cells (NK cells, CD56^+^), and B cells (CD19^+^) were positively selected using the appropriate magnetic cell separation kit (Miltenyi Biotec, Bergisch Gladbach, Germany) following the manufacturer's protocol. Use of patient material in this study was approved by the ethics committee at the University of Ulm.

### Sequence analysis

CK1δ was amplified from cDNA prepared as described above using PrimeSTAR HS DNA Polymerase. PCR was performed in a Primus 96 Plus Thermal Cycler (MWG Biotech, Ebersberg, Germany). To obtain adequate amounts of specific amplification product, a nested PCR was carried out applying the following conditions: denaturation at 98°C, annealing at 56°C, and elongation at 72°C in 35 cycles. For the first round of PCR the primers GCCCTTCACAGCAATAAGGA and CCAGAGTTCAGACCCAGGAA were used in 35 cycles. For the second round the primers GCACGACAGACTGAAGACCA and CCAGAGTTCAGACCCAGGAA were used in 20 cycles. Prior to sequencing, amplified CK1-products were separated on a 2% agarose gel and cleaned up using the peqGOLD MicroSpin gel elution kit (peqlab Biotechnologie GmbH, Erlangen, Germany). Sequencing primers for CK1δ were CCAGAGTTCAGACCCAGGAA, AGCACATCCCCTATCGTGAG, and GCACGACAGACTGAAGACCA. Sequencing of DNA was accomplished by LGC genomics (Berlin, Germany). Received CK1δ cDNA sequence data were evaluated using the database sequence for human CK1δ transcription variant 2 (accession ID: NM_139062) and the multiple sequence alignment tool ClustalW (Larkin et al., 2007; Goujon et al., 2010).

### Western blot

Cells were lysed in radio immunoprecipitation assay (RIPA) lysis buffer by incubation on ice for 30 min. Extracts were clarified by centrifugation and protein concentration was determined using the BCA protein assay (Thermo Fisher Scientific, Rockford, USA). Equal amounts of protein extracts were separated by SDS-PAGE and transferred to a PVDF membrane by semidry Western blotting. After blocking with 5% (w/v) milk the membrane was incubated with the specific antibody for CK1δ (C–18; Santa Cruz biotechnology, Heidelberg, Germany). Immunocomplexes were detected using HRP-conjugated anti-goat IgG (Santa Cruz biotechnology, Heidelberg, Germany), followed by chemiluminescence detection with SuperSignal West Dura (Thermo Fisher Scientific, Rockford, USA) and exposure to X-ray films. Densitometric quantification was performed using TINA 2.09 software. Equal loading of extracted proteins was determined by re-probing the membrane with a β-actin specific monoclonal antibody.

### Fractionation of proteins

Protein extracts for anion exchange chromatography were prepared in sucrose lysis buffer (20 mM Tris-HCl pH 7.0, 0.27 M sucrose, 1 mM EDTA, 1 mM EGTA, 1% (v/v) Triton X-100, 1 mM benzamidine, 25 μg/ml aprotinin, and 5 mM DTT) by incubation on ice for 30 min. Extracts were cleared by centrifugation and protein concentration was determined using the BCA protein assay (Thermo Fisher Scientific, Rockford, USA). Prior to column loading protein lysates were passed through a 0.4 μm filter. Fractionation of equal protein amounts was performed using an anion-exchange column (Resource Q; GE Healthcare, Chalfont St Giles, UK) attached to an ETTAN LC purifier (GE Healthcare, Chalfont St Giles, UK). Bound proteins were eluted with a linear ascending gradient between 0 and 1000 mM NaCl in fractionation buffer (50 mM Tris-HCl, pH 7.5, 1 mM EDTA, 5% (v/v) glycerol, 0.04% Brij, 1 mM benzamidine, 4 μg/ml leupeptin, and 0.1% (v/v) β-mercaptoethanol) and 250 μl of each protein fraction was collected.

### Expression and purification of glutathione S-transferase fusion proteins

Expression and purification of glutathione S-transferase (GST)-p53^1−64^ fusion protein (FP267) (Milne et al., 2001) has previously been described in detail (Knippschild et al., 1997).

### *In vitro* kinase reactions

In order to detect cellular CK1-specific kinase activity *in vitro* kinase assays were carried out using selected fractions of anion-exchange fractionated cellular protein extracts as source of kinase while the GST-p53^1−64^ fusion protein (FP267) was used as substrate. Kinase reactions were performed in kinase buffer (25 mM Tris-HCl pH 7.5, 10 mM MgCl_2_, 0.1 mM EDTA, 100 nM ATP) containing 2 μCi [γ−^32^P]-ATP per reaction. Where indicated, given concentrations of CK1-specific inhibitor compounds [IC261 (Mashhoon et al., 2000), compound 1 (Richter et al., 2014a), and compound 17 (Peifer et al., 2009)] were added. Kinase reactions were incubated at 30°C for 30 min, stopped by the addition of 5 × SDS sample buffer [250 mM Tris-HCl, pH 6.8, 25% (v/v) β-mercaptoethanol, 50% (v/v) glycerol, 10% (w/v) SDS, 0.5% (w/v) bromphenol blue], and separated by SDS-PAGE. Radioactively labeled protein bands on dried gels were visualized by autoradiography. Phosphorylated protein bands were excised and phosphate incorporation was quantified by Cherenkov counting (LS6000IC, Beckman Coulter, USA). Subsequently *in vitro* kinase assays were carried out with the CK1δ peak activity fractions of RAMOS and KM-H2 cells in presence of CK1δ specific inhibitors. For each reaction 2 μl of the inhibitor diluted in DMSO was added. Following inhibitor concentrations were used: 3 μM of IC261, 200 nM of compound 1, and 60 nM of compound 17. DMSO controls were included.

### Cell treatment and FACS analysis

For flow cytometry analysis 5 × 10^5^/ml RAMOS, KM-H2, U-H01, and DOHH-2 cells were either grown in the presence of IC261 (0.4 μM and 1.6 μM), compound 1 (2 μM and 4 μM), or compound 17 (0.5 μM and 2 μM) for 24 h and 48 h, respectively. Untreated cells and cells treated with 0.01% DMSO served as controls. At the indicated time points cells were prepared for cell cycle analysis using “Cycle Test Plus” kit (BD, San Jose, USA). Cells were stained with propidium iodide and analyzed by flow cytometry using a FACScan flow cytometer (BD bioscience, San Jose, USA) and the CellQuest software (BD, bioscience, San Jose, USA).

### Inhibitor compounds

In addition to the well-established CK1-specific inhibitor IC261 (Mashhoon et al., 2000; Cheong et al., 2011) two structurally different ATP-competitive small molecule inhibitors were used. Imidazole-derivative compound 17 has previously demonstrated increased potency and isoform selectivity for CK1δ as well as enhanced effects on cultured cells. Compound 17 is able to bind to the selectivity pocket of the CK1δ protein and therefore can be affected by certain mutations of the CK1δ gatekeeper amino acid residue (Peifer et al., 2009). Compound 1 represents a next generation CK1-specific inhibitor originating from a previously published set of benzimidazole-derived CK1-specific inhibitors (Bischof et al., 2012). By successful structure-activty relationship (SAR) based modification, a set of difluoro-dioxolo-benzoimidazole based inhibitors was developed with compound 1 showing improved inhibitory effects on CK1 isoforms δ and ε and the survival and viability of numerous tumor cell lines (Richter et al., 2014a).

## Results

### Analysis of CK1δ mRNA and protein levels in established lymphoma cell lines

Several studies indicate that deregulated expression and/or activity of CK1 is associated with tumorigenesis in a number of malignancies (Inuzuka et al., 2010; Elyada et al., 2011; Knippschild et al., 2014). However, for human malignant lymphoma the impact of CK1δ on tumor development or progression has not been systematically investigated so far. In order to determine CK1δ expression levels, we first conducted quantitative reverse-transcription PCR (qRT-PCR). CK1δ mRNA was found in all 18 cell lines investigated. Both PMBL (mediastinal large B cell lymphoma) cell lines, MedB-1 and KARPAS-1066P, showed about twofold higher amounts of CK1δ mRNA than the other lymphoma cell lines included in our study (Figure 1A). CK1δ protein expression was quantified by Western blotting analysis and could be detected in all tested cell lines of the various lymphoma entities. In contrast to the mRNA findings, PMBL cell lines did not show elevated CK1δ protein expression, whereas the cHL (classical Hodgkin lymphoma) group and the single tested T-ALL (T cell acute lymphoblastic lymphoma) cell line displayed slightly increased CK1δ protein levels in comparison to the other lymphoma entities (Figure 1B).

### CK1δ-specific kinase activity differs in established lymphoma cell lines

**Figure 2:**
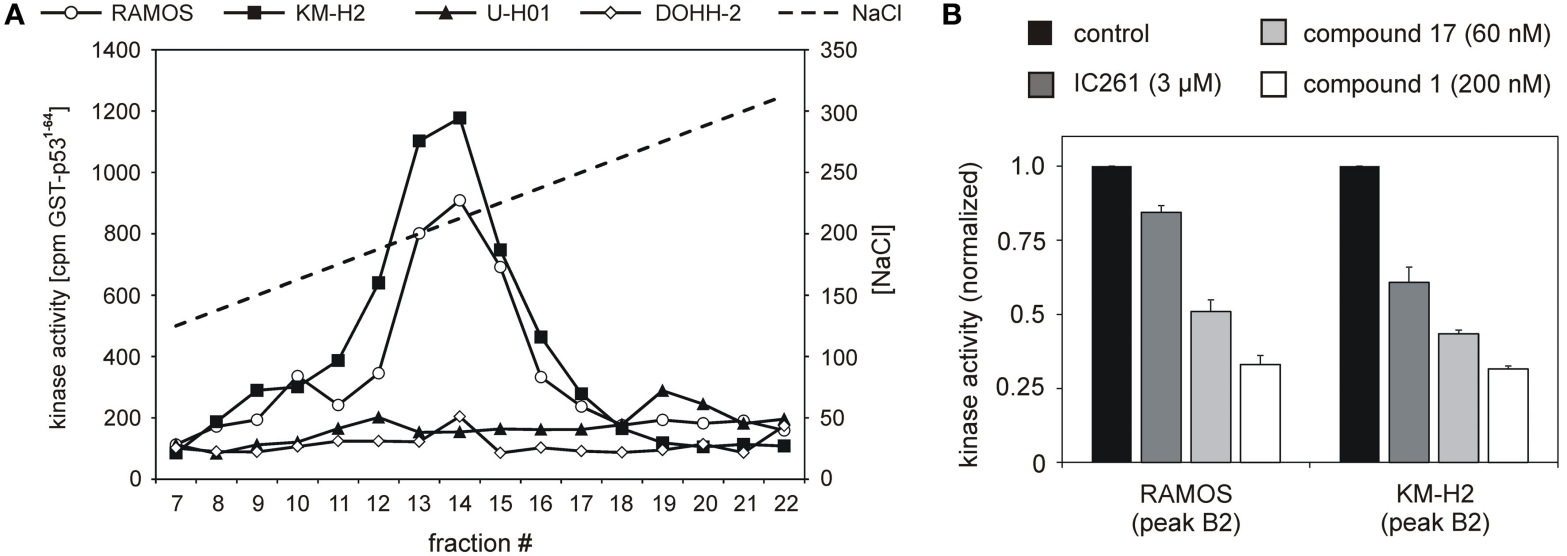
**Cell lines RAMOS, KM-H2, U-H01, and DOHH-2 show differences in CK1-specific kinase activity. (A)** Protein extracts from RAMOS, KM-H2, U-H01, and DOHH-2 cells were ion-exchange fractionated and resulting fractions were used to phosphorylate GST-p53^1-64^. Kinase reactions were separated in SDS-PAGE and phosphate incorporation into the substrate was quantified by Cherenkov counting. While cell lines RAMOS and KM-H2 show high CK1-specific activity no comparable kinase peak could be detected for U-H01 and DOHH-2 cells. **(B)** Peak kinase activity was assayed in presence of the CK1-specific inhibitors IC261 (Mashhoon et al., 2000), compound 1 (Richter et al., 2014a), and compound 17 (Peifer et al., 2009). Strong inhibition of peak kinase activity most of all by the CK1δ-specific compound 1 clearly confirms contribution of CK1δ. Experiments were performed in triplicate. Data are presented as mean values, error bars indicate standard error of the mean (SEM).

CK1 activity is tightly regulated by various cellular mechanisms. Having demonstrated CK1δ expression, we therefore tested CK1δ-specific kinase activity in selected lymphoma cell lines: the two classical HL lines KM-H2 and U-H01, the diffuse large B cell lymhoma line DOHH-2, and the Burkitt lymphoma line RAMOS. Equal amounts of protein extracts were fractionated by ion-exhange chromatography in order to separate CK1 from other protein kinases as well as cellular modulators of kinase acitvity like activating/inhibiting binding partners and phosphatases. Resulting fractions were checked for CK1 kinase activity using the substrate GST-p53^1−64^. Strong CK1-specific activity could be detected in extracts of KM-H2 and RAMOS cells while U-H01 and DOHH-2 cells only showed weak detectable activity (Figure 2A). In order to confirm a major contribution of CK1δ to the detected activity in RAMOS and KM-H2 cells, kinase activity in the peak fractions was assayed in the presence of different CK1-specific inhibitors, including IC261 (Mashhoon et al., 2000), compound 1 (Richter et al., 2014a), and compound 17 (Peifer et al., 2009). For both cell lines kinase activity in the peak fractions could be inhibited by all three substances. At least for compounds 1 and 17 strong CK1δ isoform-selectivity has been reported. Strongest activity-reduction of up to 70% was demonstrated with compound 1, confirming, that the detected activity peak indeed mainly was caused by CK1δ (Figure 2B).

### Sequence analysis of CK1δ in established lymphoma cell lines

**Figure 3:**
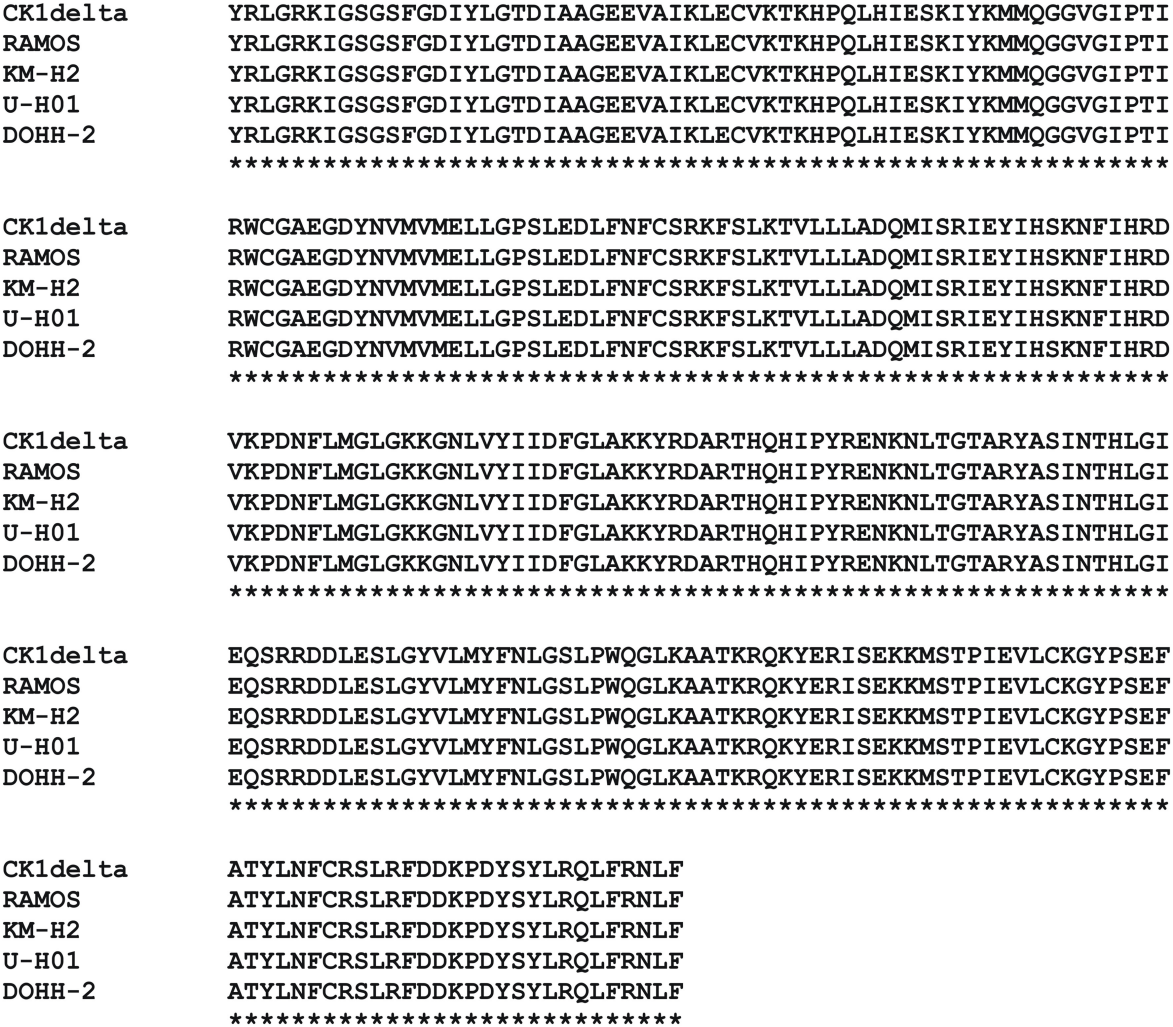
**Protein sequence alignment of CK1δ kinase domain amplified from cell lines RAMOS, KM-H2, U-H01, and DOHH-2**. Messenger RNA from cell lines RAMOS, KM-H2, U-H01, and DOHH-2 was isolated, transcribed into cDNA, and the CK1δ coding sequence was amplified as described in Material and Methods. DNA sequences obtained from reactions using primers listed in Material and Methods were translated into protein sequences and the alignment was generated using ClustalW 2.1 (Larkin et al., 2007; Goujon et al., 2010).

Published data indicate that gene mutations may enhance the oncongenic potential of CK1δ (Tsai et al., 2007; Schittek and Sinnberg, 2014). Therefore, we screended for CK1δ coding region mutations by direct sequencing of RT-PCR-products. All 18 lymphoma cell lines were found to express wildtype CK1δ (Figure 3).

### CK1-specific inhibitors show different effects on cell cycle distribution of various lymphoma cell lines

**Figure 4:**
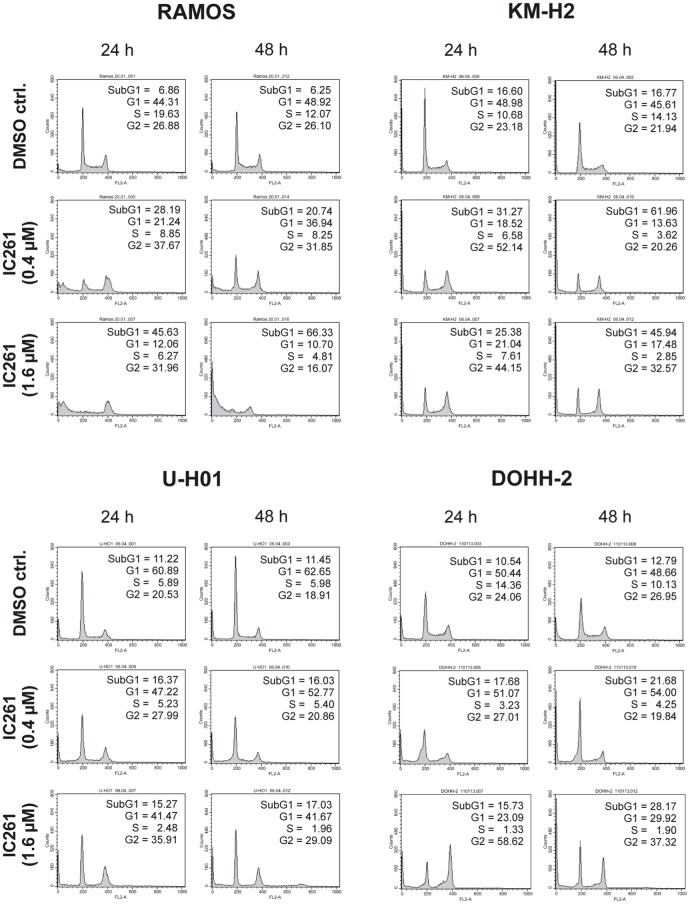
**Cell cycle analyses of RAMOS, KM-H2, U-H01, and DOHH-2 cells after treatment with the CK1δ/ε-specific inhibitor IC261**. Cells were treated with different concentrations of IC261 for 24 h and 48 h and stained with propidium iodide in order to perform cell cycle analysis as described in Material and Methods. DMSO treated cells served as control. Each 20.000 cells in two replicates per condition were counted. One representative graph is shown. The determined fractions of cells in various phases of the cell cycle are given as percent values (%). Flow cytometry plots show the number of cells (y-axis) with each observed intensity of propidium iodide DNA staining (x-axis). DMSO treated cells showed a normal cell cycle distribution of asynchronously proliferating cells. Treatment with IC261 led to an increase of dead RAMOS cells, whereas in KM-H2, U-H01, and DOHH-2 cells an increase of G2 arrested cells could be observed.

**Figure 5:**
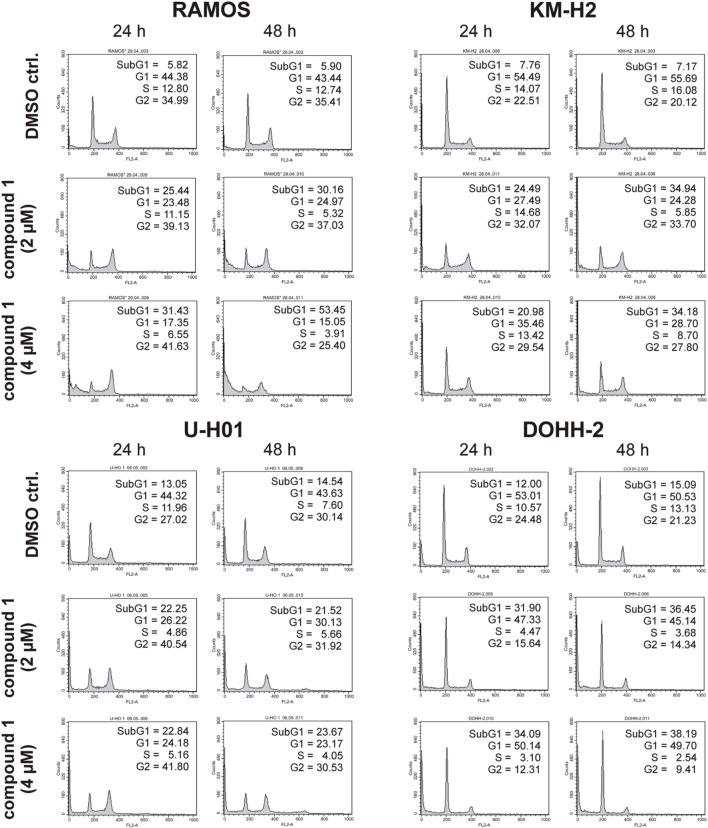
**Cell cycle analyses of RAMOS, KM-H2, U-H01, and DOHH-2 cells after treatment with the CK1δ-specific inhibitor compound 1**. Cells were treated with different concentrations of compound 1 for 24 h and 48 h and stained with propidium iodide in order to perform cell cycle analysis as described in Material and Methods. DMSO treated cells served as control. Each 20.000 cells in two replicates per condition were counted. One representative graph is shown. The determined fractions of cells in various phases of the cell cycle are given as percent values (%). Flow cytometry plots show the number of cells (y-axis) with each observed intensity of propidium iodide DNA staining (x-axis). DMSO treated cells showed a normal cell cycle distribution of asynchronously proliferating cells. Treatment with compound 1 led to an increase of RAMOS, KM-H2, and U-H01 cells in G2 phase. Additionally, RAMOS cells accumulate in Sub G1 phase as well. No obvious effects could be observed in DOHH-2 cells.

**Figure 6:**
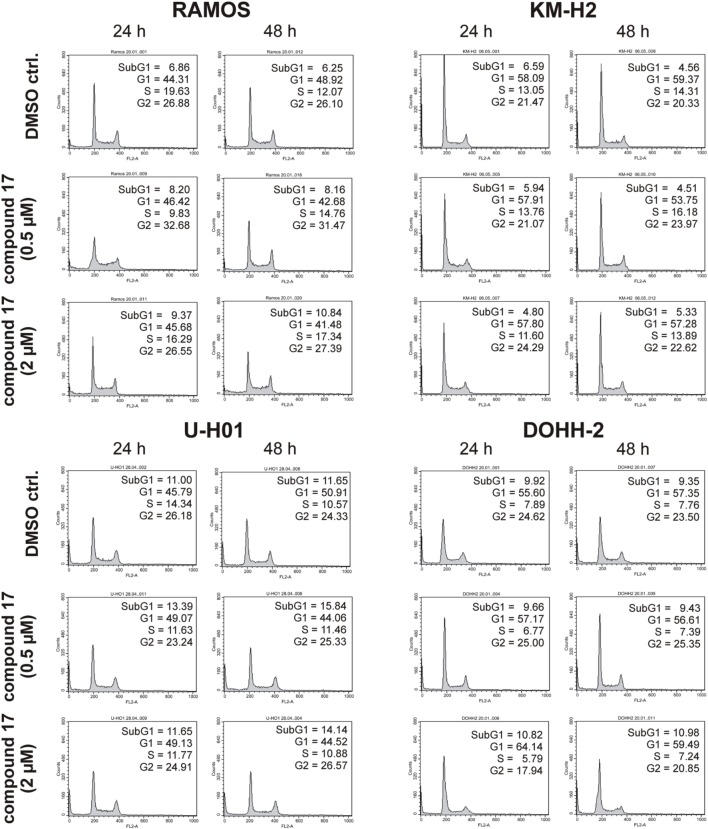
**Cell cycle analyses of RAMOS, KM-H2, U-H01, and DOHH-2 cells after treatment with the CK1δ-specific inhibitor compound 17**. Cells were treated with different concentrations of compound 17 for 24 h and 48 h and stained with propidium iodide in order to perform cell cycle analysis as described in Material and Methods. DMSO treated cells served as control. Each 20.000 cells in two replicates per condition were counted. One representative graph is shown. The determined fractions of cells in various phases of the cell cycle are given as percent values (%). Flow cytometry plots show the number of cells (y-axis) with each observed intensity of propidium iodide DNA staining (x-axis). DMSO treated cells showed a normal cell cycle distribution of asynchronously proliferating cells. Treatment with compound 17 only led to minor effects on all tested cell lines.

Since expression of wildtype CK1δ could be detected in all analyzed cell lines and significant CK1δ-specific kinase activity could be detected at least in RAMOS and KM-H2 cells, the influence of CK1-specific inhibitors on cell cycle distribution was determined for selected lymphoma cell lines. Treatment with the CK1δ/ε-specific inhibitor IC261 showed obvious effects on RAMOS, KM-H2, and DOHH-2 cells by either increasing the amount of dead cells in case of RAMOS cells or by leading to cell cycle arrest in G2 phase for an increased number of KM-H2 and DOHH-2 cells. In contrast IC261 treatment only led to minor effects on U-H01 cells (Figure 4). While compound 17 showed no obvious effects on the tested cell lines treatment with only low concentrations of compound 1 resulted in an increase of dead cells already after 24 h (Figures 5, 6). Except for RAMOS these effects of compound 1 could not be potentiated but remained stable while treatment duration or inhibitor concentration was increased. All compounds showed most obvious effects on RAMOS cells after 24 h which could be enhanced by either increasing inhibitor concentration or treatment duration to 48 h.

### CK1δ mRNA expression levels in lymphoma tissues correspond to tumor cell lines but differ from non-neoplastic lymphoid cells

**Figure 7:**
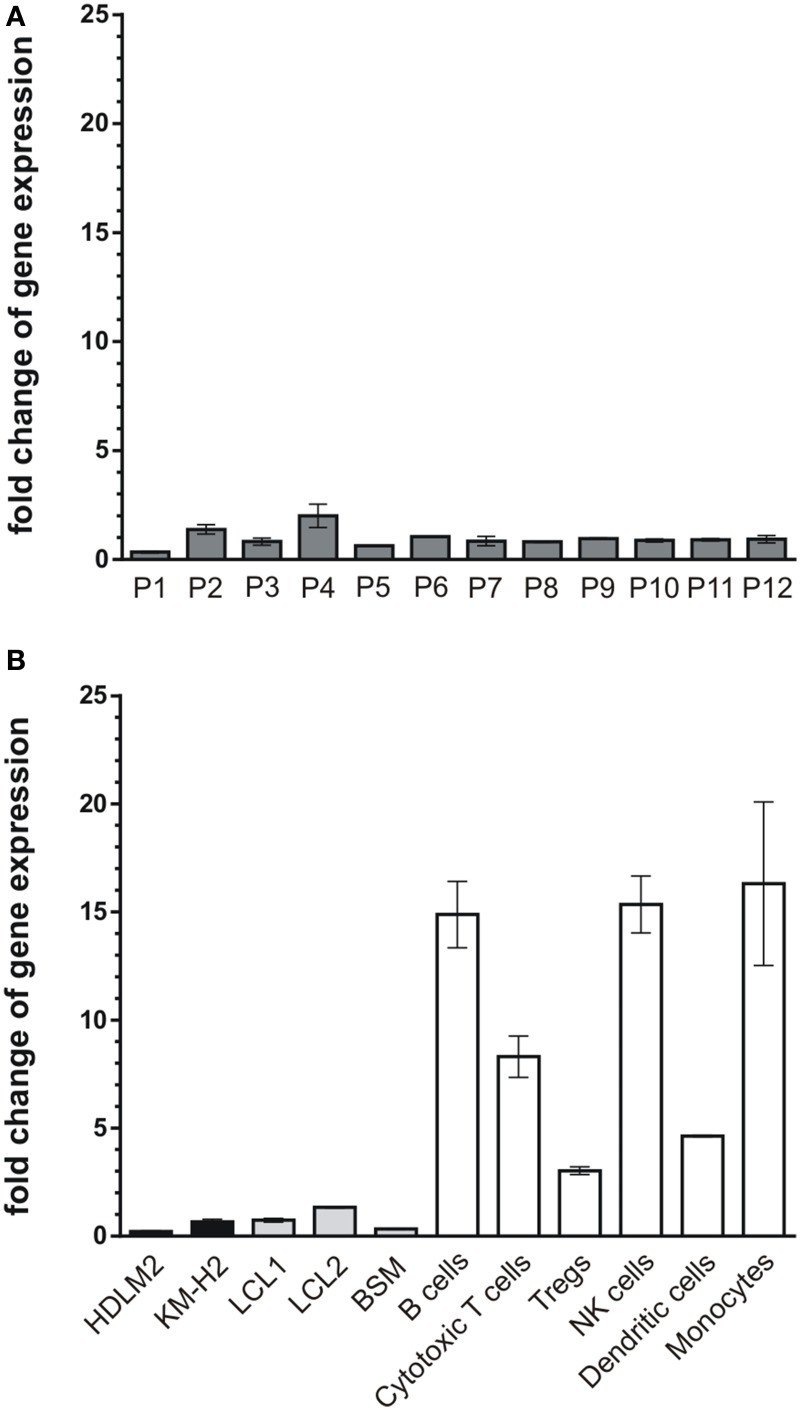
**mRNA expression levels of CK1δ in diffuse large B cell lymphoma tissues compared to lymphocytes from healthy donors**. Comparison of CK1δ mRNA expression levels between **(A)** tumors of lymphoid tissues from 12 patients (P1–P12), **(B)** the established lymphoma cell lines HDLM2 and KM-H2 as well as the EBV-transformed lymphoblastoid B cells LCL1, LCL2, and BSM, and lymphocytes and other blood cells of healthy donors by quantitative real-time PCR as described in Materials and Methods. Results clearly demonstrate decreased mRNA expression levels of CK1δ in lymphoid tissues, lymphoma cell lines and EBV-transformed lymphoblastoid B cells compared to lymphocytes from healthy donors. Experiments were performed in duplicate. Data are presented as mean values of relative gene expression compared to HPRT mRNA levels, error bars indicate standard deviation (SD).

So far, our results demonstrate that CK1-specific inhibitors differently affect the growth of various lymphoma cell lines. Their use in novel therapeutic concepts could be considered, especially if alterations in the expression levels of CK1δ in tumor tissue are detectable. Therefore, we now analyzed CK1δ mRNA expression in 12 tissue samples of diffuse large B cell lymphoma, which is the most prevalent aggressive lymphoma type. We also measured the expression levels in EBV-immortalized lymphoid cells, and non-neoplastic lymphoid cell populations from healthy donors. In the tested tumor tissue samples the expression levels of CK1δ varied up to 5.9-fold (Figure 7A, Table 1). Similarly, the expression levels of CK1δ among blood cells and lymphocytes from healthy donors also varied up to 5.4-fold (Figure 7B, Table 1). However, CK1δ expression in non-neoplastic blood cells (i.e., B cells, Tregs, cytotoxic T cells, and monocytes) were markedly increased compared to expression levels in tumor samples, lymphoma cell lines, and EBV-transformed lymphoblastoid B cells (Figures 7A,B).

**Table 1:** **CK1δ expression levels in tumors from lymphoid tissue, established lymphoma cell lines, EBV-immortalized lymphoblastoid B cells, and peripheral blood cell populations of healthy donors**.

**Sample**	**Fold change expression of CK1δ**
P1	0.34±0.06
P2	1.39±0.30
P3	0.82±0.23
P4	2.00±0.76
P5	0.64±0.01
P6	1.05±0.01
P7	0.85±0.30
P8	0.81±0.03
P9	0.96±0.05
P10	0.88±0.09
P11	0.91±0.07
P12	0.94±0.23
HDLM2	0.22±0.04
KM-H2	0.66±0.16
LCL1	0.74±0.12
LCL2	1.34±0.05
BSM	0.33±0.02
B cells	14.88±2.17
Cytotoxic T cells	8.30±1.35
Tregs	3.03±0.26
NK cells	15.35±1.87
Dendritic cells	4.64±0.05
Monocytes	16.31±5.34

*Values represent fold change of relative CK1δ expression ± SD compared to HPRT mRNA levels*.

## Discussion

Protein kinases of the CK1 family are involved in essential cellular processes and CK1 dysregulation can be associated with the pathogenesis of certain diseases as well as tumorigenesis (Knippschild et al., 2014). Recently, CK1α was suggested as therapeutic target for innovative strategies to treat malignancies arising from the hematopoietic system like multiple myeloma (Hu et al., 2014), acute myeloid leukemia (Jaras et al., 2014), or del(5q) myelodysplastic syndrome (Schneider et al., 2014). In the present study, we demonstrate the therapeutic potential of CK1-specific kinase inhibition by treating established lymphoma cell lines with CK1-specific small molecule inhibitors. In order to characterize the CK1-specific status and to estimate the expected treatment effects, we first analyzed CK1δ expression and mutation status in 18 cell lines covering a broad spectrum of lymphoid neoplasms. In all cell lines CK1δ mRNA and protein were found to be expressed strongly and at a similar level (except mRNA expression in PMBL cell lines) pointing to essential functions of CK1 in the regulation of apoptosis, NFκ B, and Wnt-related signaling (Cruciat, 2014; Knippschild et al., 2014). Previous studies carried out in a murine model system already detected increased expression of CK1δ in hyperplastic B cell follicles and progressed B cell lymphomas in p53-deficient mice (Maritzen et al., 2003).

Mutations in the coding sequences of central signal transduction molecules play a pivotal role in tumorigenesis. In a number of genes, somatic mutations are quite common in malignant lymphoma and can even be characteristic for certain lymphoma entities (Kuppers, 2005). Mutations in the CK1δ protein may lead to altered binding and potency of certain inhibitor molecules (Peifer et al., 2009; Richter et al., 2014b). Therefore, mutation analysis is essential when determining the therapeutic potential of CK1-specific inhibitor compounds. Remarkably however, the analyzed human lymphoma cell lines without exception showed an unmutated state of the CK1δ coding sequence. This finding may point to an essential need for unmutated and functional CK1δ in signaling pathways required for intact lymphoid neoplasm cell function. Further analysis would be needed to validate this conclusion. An important mechanism leading to mutations in malignant lymphoma is cytidine deamination by activation induced cytidine deaminase (AID) and the subsequent incorrect repair of the affected base. Since chromatin-immunoprecipitation studies in murine B-lymphocytes revealed that AID is not able to substantially bind to CK1δ DNA sequence (Yamane et al., 2011), this could provide one explanation for the absence of CK1δ mutations while some of the analyzed cell lines carry mutations in multiple genes (Kuppers, 2005; Mader et al., 2007).

For further analyses on the role of CK1δ in human lymphoma cell lines we concentrated on four cell lines, representing classical Hodgkin lymphoma (KM-H2 and U-H01), diffuse large B cell lymphoma (DOHH-2) and Burkitt lymphoma (RAMOS). Although all four cell lines share rather similar CK1δ expression levels, differences in the cellular kinase activity of CK1δ could be detected. Using the established CK1-specific small molecule inhibitor IC261 and two structurally independent CK1δ-specific inhibitors (Peifer et al., 2009; Richter et al., 2014a) the kinase activity detected using substrate GST-p53^1−64^ (Knippschild et al., 1997) could clearly be assigned to CK1δ. Cell line specific differences might be explained by posttranslational modifications of CK1δ and have already been reported in previous studies (Maritzen et al., 2003; Giamas et al., 2007). Mechanisms that regulate CK1δ activity are intramolecular autophosphorylation and phosphorylation through other cellular kinases (Rivers et al., 1998; Gietzen and Virshup, 1999; Giamas et al., 2007; Bischof et al., 2013).

In all four cell lines, FACS analyses demonstrated changes in cell cycle distribution after treatment with CK1δ-specific inhibitor compounds. Due to reduced stability and solubility of inhibitor compounds 1 and 17 much higher concentrations of these compounds have to be used for the treatment of cultured cells when being compared to *in vitro* application and to the use of IC261. The observed effects are dependent on inhibitor concentration, cell line, and duration of inhibitor exposure. Cell cycle arrest, as observed for some cell lines being treated with IC261 and compound 1, can be explained by CK1 effects on microtubule dynamics and chromosome segregation (Behrend et al., 2000; Stoter et al., 2014). However, Cheong and colleagues meanwhile demonstrated, that IC261 could have a similar effect on cellular microtubule dynamics as the spindle poison colchicine (Cheong et al., 2011). In order to exclude unspecific effects, we therefore performed all experiments with three structurally different CK1-inhibitors. Previous studies showed that the status of the tumor suppressor p53 plays a central role for the effects induced by inhibitor IC261. While treatment of wildtype p53 expressing cells with CK1-inhibitors leads to postmitotic arrest or apoptosis, the treatment of cells expressing mutated or no p53 at all is followed by endoreduplication (Behrend et al., 2000; Stoter et al., 2005). The present study is in partial accordance to these reports. Cell lines KM-H2 and DOHH-2 (wt p53, Drakos et al., 2007; Wang et al., 2011) showed increased apoptosis upon treatment with IC261 and compound 1. Treatment with IC261, however, also arrested an increased number of KM-H2 and DOHH-2 cells in G2-phase of the cell cycle. Albeit expressing mutated p53 (Farrell et al., 1991), RAMOS cells showed a remarkable increase of apoptotic cells after treatment with the CK1-specific inhibitors. In DOHH-2 cells the chromosomal translocation t(14:18) results in overexpression of Bcl-2 which is associated with chemoresistance (Tsujimoto et al., 1984; Kluin-Nelemans et al., 1991; Reed, 2008). The anti-apoptotic effect of Bcl2 may explain the low level of apoptosis induction that could be achieved in DOHH-2 treated with CK1-specific inhibitors. Similarly, the reduced reactivity to inhibitor treatment of cell line U-H01 could be explained by mutation of the phosphatase PTPN1 which protects the cells from apoptosis and therefore provides an advantage for cell survival (Knecht et al., 2010). Generally, the low activity of CK1δ detected in U-H01 and DOHH-2 provides another reason why the observed effects are lower than in KM-H2 or RAMOS, two cell lines showing high cellular CK1δ kinase activity.

After having demonstrated significant effects of CK1-specific inhibitors on established lymphoma cell lines, the mRNA expression levels of CK1δ in primary lymphoid tumor tissue, EBV-immortalized lymphoid cells, and blood cells from healthy donors were compared. While the variation of CK1δ mRNA levels was nearly the same in primary tumor tissue and normal blood cells, CK1δ mRNA levels in general were remarkably low in all tested samples in comparison to blood cells from healthy donors. Cell lines HDLM2 and KM-H2, which were also included in this comparison, showed similarly low CK1δ mRNA levels as the analyzed primary tumor tissue samples. Therefore, these data provide further evidence that these cell lines may be used as a model for the validation of CK1-specific inhibitors in new lymphoma treatment strategies.

As a conclusion, its multiple functions may qualify CK1 as a new potential target molecule to treat lymphoid neoplasms. The results of the present study suggest for the first time that inhibition of CK1δ could have adjuvant effects in the therapy of malignant lymphoma. However, these effects are strongly dependent on the cellular background of the individual lymphoma cell population and for future application in novel concepts of personalized medicine still large efforts have to be made to increase solubility and selectivity of the inhibitor compounds as well as to improve their safe delivery to the target tissue to avoid severe side effects.

## Author contributions

Project work was designed by UK and FL. Experimental work was performed by BW, FO, JR, JB, PX, and TB. Data analysis and interpretation was done by BW, FO, JR, JB, PX, TB, FL, and UK. All Authors (BW, FO, JR, JB, PX, TB, FL, and UK) were involved in writing passages of the present manuscript and participated in final approval and revision. Figures were created by BW, FO, JR, and JB.

### Conflict of interest statement

The authors declare that the research was conducted in the absence of any commercial or financial relationships that could be construed as a potential conflict of interest.

## References

[B1] Aalipour A. Advani R. H. (2014). Bruton's tyrosine kinase inhibitors and their clinical potential in the treatment of B-cell malignancies: focus on ibrutinib. Ther. Adv. Hematol. 5, 121–133. 10.1177/2040620714539906 25360238 PMC4212313

[B2] Behrend L. Milne D. M. Stoter M. Deppert W. Campbell L. E. Meek D. W.. (2000). IC261, a specific inhibitor of the protein kinases casein kinase 1-delta and -epsilon, triggers the mitotic checkpoint and induces p53-dependent postmitotic effects. Oncogene 19, 5303–5313. 10.1038/sj.onc.1203939 11103931

[B2a] Bischof J. Leban J. Zaja M. Grothey A. Radunsky B. Othersen O.. (2012). 2-Benzamido-N-(1H-benzo[d]imidazol-2-yl)thiazole-4-carboxamide derivatives as potent inhibitors of CK1δ/ϵ. Amino Acids 43, 1577–1591. 10.1007/s00726-012-1234-x 22331384 PMC3448056

[B3] Bischof J. Randoll S. J. Sussner N. Henne-Bruns D. Pinna L. A. Knippschild U. (2013). CK1delta kinase activity is modulated by Chk1-mediated phosphorylation. PLoS ONE 8:e68803. 10.1371/journal.pone.0068803 23861943 PMC3701638

[B4] Borowitz M. J. Chan J. K. C. (2008). B lymphoblastic leukaemia/lymphoma, not otherwise specified, in WHO Classification of Tumours of Haematopoietic and Lymphoid Tissues, eds Swerdlow S. H. Campo E. Harris N. L. Jaffe E. S. Pileri S. A. Stein H. Thiele J. Vardiman J. W. (Lyon: IARC), 168–170.

[B5] Burzio V. Antonelli M. Allende C. C. Allende J. E. (2002). Biochemical and cellular characteristics of the four splice variants of protein kinase CK1alpha from zebrafish (Danio rerio). J. Cell. Biochem. 86, 805–814. 10.1002/jcb.10263 12210746

[B6] Cheong J. K. Hung N. T. Wang H. Tan P. Voorhoeve P. M. Lee S. H.. (2011). IC261 induces cell cycle arrest and apoptosis of human cancer cells via CK1delta/varepsilon and Wnt/beta-catenin independent inhibition of mitotic spindle formation. Oncogene 30, 2558–2569. 10.1038/onc.2010.627 21258417 PMC3109269

[B7] Cheong J. K. Virshup D. M. (2011). Casein kinase 1: complexity in the family. Int. J. Biochem. Cell Biol. 43, 465–469. 10.1016/j.biocel.2010.12.004 21145983

[B8] Cruciat C. M. (2014). Casein kinase 1 and Wnt/beta-catenin signaling. Curr. Opin. Cell Biol. 31C, 46–55. 10.1016/j.ceb.2014.08.003 25200911

[B9] Diehl V. Kirchner H. H. Schaadt M. Fonatsch C. Stein H. Gerdes J.. (1981). Hodgkin's disease: establishment and characterization of four *in vitro* cell lies. J. Cancer Res. Clin. Oncol. 101, 111–124. 7276066 10.1007/BF00405072 PMC12253201

[B10] Dietrich S. Glimm H. Andrulis M. Von Kalle C. Ho A. D. Zenz T. (2012). BRAF inhibition in refractory hairy-cell leukemia. N. Engl. J. Med. 366, 2038–2040. 10.1056/NEJMc1202124 22621641

[B11] Drakos E. Thomaides A. Medeiros L. J. Li J. Leventaki V. Konopleva M.. (2007). Inhibition of p53-murine double minute 2 interaction by nutlin-3A stabilizes p53 and induces cell cycle arrest and apoptosis in Hodgkin lymphoma. Clin. Cancer Res. 13, 3380–3387. 10.1158/1078-0432.CCR-06-2581 17545546

[B12] Drexler H. G. Gaedicke G. Lok M. S. Diehl V. Minowada J. (1986). Hodgkin's disease derived cell lines HDLM-2 and L-428: comparison of morphology, immunological and isoenzyme profiles. Leuk. Res. 10, 487–500. 3713248 10.1016/0145-2126(86)90084-6

[B13] Elyada E. Pribluda A. Goldstein R. E. Morgenstern Y. Brachya G. Cojocaru G.. (2011). CKIalpha ablation highlights a critical role for p53 in invasiveness control. Nature 470, 409–413. 10.1038/nature09673 21331045

[B14] Epstein A. L. Levy R. Kim H. Henle W. Henle G. Kaplan H. S. (1978). Biology of the human malignant lymphomas. IV. Functional characterization of ten diffuse histiocytic lymphoma cell lines. Cancer 42, 2379–2391. 214220 10.1002/1097-0142(197811)42:5<2379::aid-cncr2820420539>3.0.co;2-4

[B15] Farrell P. J. Allan G. J. Shanahan F. Vousden K. H. Crook T. (1991). p53 is frequently mutated in Burkitt's lymphoma cell lines. EMBO J. 10, 2879–2887. 1915267 10.1002/j.1460-2075.1991.tb07837.x PMC452998

[B16] Fu Z. Chakraborti T. Morse S. Bennett G. S. Shaw G. (2001). Four casein kinase I isoforms are differentially partitioned between nucleus and cytoplasm. Exp. Cell Res. 269, 275–286. 10.1006/excr.2001.5324 11570820

[B17] Giamas G. Hirner H. Shoshiashvili L. Grothey A. Gessert S. Kuhl M.. (2007). Phosphorylation of CK1delta: identification of Ser370 as the major phosphorylation site targeted by PKA *in vitro* and *in vivo*. Biochem. J. 406, 389–398. 10.1042/BJ20070091 17594292 PMC2049039

[B18] Gietzen K. F. Virshup D. M. (1999). Identification of inhibitory autophosphorylation sites in casein kinase I epsilon. J. Biol. Chem. 274, 32063–32070. 10542239 10.1074/jbc.274.45.32063

[B19] Goujon M. Mcwilliam H. Li W. Valentin F. Squizzato S. Paern J.. (2010). A new bioinformatics analysis tools framework at EMBL-EBI. Nucleic Acids Res. 38, W695–W699. 10.1093/nar/gkq313 20439314 PMC2896090

[B20] Green C. L. Bennett G. S. (1998). Identification of four alternatively spliced isoforms of chicken casein kinase I alpha that are all expressed in diverse cell types. Gene 216, 189–195. 10.1016/S0378-1119(98)00291-1 9766967

[B21] Halder T. M. Bluggel M. Heinzel S. Pawelec G. Meyer H. E. Kalbacher H. (2000). Defensins are dominant HLA-DR-associated self-peptides from CD34(-) peripheral blood mononuclear cells of different tumor patients (plasmacytoma, chronic myeloid leukemia). Blood 95, 2890–2896. 10779436

[B22] Harris N. L. Campo E. Jaffe E. S. Pileri S. A. Stein H. Swerdlow S. H. (2008). Introduction to the WHO Classification of Tumours of Haematopoietic and Lymphoid Tissues. Lyon: IARC.

[B23] Hu Y. Song W. Cirstea D. Lu D. Munshi N. C. Anderson K. C. (2014). CSNK1alpha1 mediates malignant plasma cell survival. Leukemia 29, 474–482. 10.1038/leu.2014.202 24962017 PMC4276736

[B24] Hurwitz R. Hozier J. Lebien T. Minowada J. Gajl-Peczalska K. Kubonishi I.. (1979). Characterization of a leukemic cell line of the pre-B phenotype. Int. J. Cancer 23, 174–180. 83966 10.1002/ijc.2910230206

[B25] Inuzuka H. Tseng A. Gao D. Zhai B. Zhang Q. Shaik S.. (2010). Phosphorylation by casein kinase I promotes the turnover of the Mdm2 oncoprotein via the SCF(beta-TRCP) ubiquitin ligase. Cancer Cell 18, 147–159. 10.1016/j.ccr.2010.06.015 20708156 PMC2923652

[B26] Jaffe E. S. Harris N. L. Stein H. Vardiman J. W. (2001). World Health Organization Classification of Tumours: Pathology and Genetics of Tumours of Haematopoietic and Lymphoid Tissues. Lyon: IARC Press.

[B27] Jaras M. Miller P. G. Chu L. P. Puram R. V. Fink E. C. Schneider R. K.. (2014). Csnk1a1 inhibition has p53-dependent therapeutic efficacy in acute myeloid leukemia. J. Exp. Med. 211, 605–612. 10.1084/jem.20131033 24616378 PMC3978274

[B28] Kamesaki H. Fukuhara S. Tatsumi E. Uchino H. Yamabe H. Miwa H.. (1986). Cytochemical, immunologic, chromosomal, and molecular genetic analysis of a novel cell line derived from Hodgkin's disease. Blood 68, 285–292. 3013343

[B29] Kanzler H. Kuppers R. Hansmann M. L. Rajewsky K. (1996). Hodgkin and Reed-Sternberg cells in Hodgkin's disease represent the outgrowth of a dominant tumor clone derived from (crippled) germinal center B cells. J. Exp. Med. 184, 1495–1505. 8879220 10.1084/jem.184.4.1495 PMC2192840

[B30] Klein E. Klein G. Nadkarni J. S. Nadkarni J. J. Wigzell H. Clifford P. (1968). Surface IgM-kappa specificity on a Burkitt lymphoma cell *in vivo* and in derived culture lines. Cancer Res. 28, 1300–1310. 4174339

[B31] Klein G. Giovanella B. Westman A. Stehlin J. S. Mumford D. (1975). An EBV-genome-negative cell line established from an American Burkitt lymphoma; receptor characteristics. EBV infectibility and permanent conversion into EBV-positive sublines by *in vitro* infection. Intervirology 5, 319–334. 181343 10.1159/000149930

[B32] Kluin-Nelemans H. C. Limpens J. Meerabux J. Beverstock G. C. Jansen J. H. De Jong D.. (1991). A new non-Hodgkin's B-cell line (DoHH2) with a chromosomal translocation t(14;18)(q32;q21). Leukemia 5, 221–224. 1849602

[B33] Knecht H. Bruderlein S. Wegener S. Lichtensztejn D. Lichtensztejn Z. Lemieux B.. (2010). 3D nuclear organization of telomeres in the Hodgkin cell lines U-HO1 and U-HO1-PTPN1: PTPN1 expression prevents the formation of very short telomeres including “t-stumps.” BMC Cell Biol. 11:99. 10.1186/1471-2121-11-99 21144060 PMC3018409

[B34] Knippschild U. Kruger M. Richter J. Xu P. Garcia-Reyes B. Peifer C.. (2014). The CK1 family: contribution to cellular stress response and its role in carcinogenesis. Front. Oncol. 4:96. 10.3389/fonc.2014.00096 24904820 PMC4032983

[B35] Knippschild U. Milne D. M. Campbell L. E. Demaggio A. J. Christenson E. Hoekstra M. F.. (1997). p53 is phosphorylated *in vitro* and *in vivo* by the delta and epsilon isoforms of casein kinase 1 and enhances the level of casein kinase 1 delta in response to topoisomerase-directed drugs. Oncogene 15, 1727–1736. 9349507 10.1038/sj.onc.1201541

[B36] Knippschild U. Wolff S. Giamas G. Brockschmidt C. Wittau M. Wurl P. U.. (2005). The role of the casein kinase 1 (CK1) family in different signaling pathways linked to cancer development. Onkologie 28, 508–514. 10.1159/000087137 16186692

[B37] Kuppers R. (2005). Mechanisms of B-cell lymphoma pathogenesis. Nat. Rev. Cancer 5, 251–262. 10.1038/nrc1589 15803153

[B38] Larkin M. A. Blackshields G. Brown N. P. Chenna R. Mcgettigan P. A. Mcwilliam H.. (2007). Clustal W and Clustal X version 2.0. Bioinformatics 23, 2947–2948. 10.1093/bioinformatics/btm404 17846036

[B39] Leoncini L. Raphael M. Stein H. Harris N. L. Jaffe E. S. Kluin P. M. (2008). Burkitt lymphoma, in WHO Classification of Tumours of Haematopoietic and Lymphoid Tissues, eds Swerdlow S. H. Campo E. Harris N. L. Jaffe E. S. Pileri S. A. Stein H. Thiele J. Vardiman J. W. (Lyon: IARC), 262–264.

[B40] Mader A. Bruderlein S. Wegener S. Melzner I. Popov S. Muller-Hermelink H. K.. (2007). U-HO1, a new cell line derived from a primary refractory classical Hodgkin lymphoma. Cytogenet. Genome Res. 119, 204–210. 10.1159/000112062 18253030

[B41] Maritzen T. Lohler J. Deppert W. Knippschild U. (2003). Casein kinase I delta (CKIdelta) is involved in lymphocyte physiology. Eur. J. Cell Biol. 82, 369–378. 10.1078/0171-9335-00323 12924632

[B42] Mashhoon N. Demaggio A. J. Tereshko V. Bergmeier S. C. Egli M. Hoekstra M. F.. (2000). Crystal structure of a conformation-selective casein kinase-1 inhibitor. J. Biol. Chem. 275, 20052–20060. 10.1074/jbc.M001713200 10749871

[B43] Menon M. P. Pittaluga S. Jaffe E. S. (2012). The histological and biological spectrum of diffuse large B-cell lymphoma in the World Health Organization classification. Cancer J. 18, 411–420. 10.1097/PPO.0b013e31826aee97 23006945 PMC3458515

[B44] Milne D. M. Looby P. Meek D. W. (2001). Catalytic activity of protein kinase CK1 delta (casein kinase 1delta) is essential for its normal subcellular localization. Exp. Cell Res. 263, 43–54. 10.1006/excr.2000.5100 11161704

[B45] Moller P. Bruderlein S. Strater J. Leithauser F. Hasel C. Bataille F.. (2001). MedB-1, a human tumor cell line derived from a primary mediastinal large B-cell lymphoma. Int. J. Cancer 92, 348–353. 10.1002/ijc.1211 11291070

[B46] Nacheva E. Dyer M. J. Metivier C. Jadayel D. Stranks G. Morilla R.. (1994). B-cell non-Hodgkin's lymphoma cell line (Karpas 1106) with complex translocation involving 18q21.3 but lacking BCL2 rearrangement and expression. Blood 84, 3422–3428. 7949096

[B47] Peifer C. Abadleh M. Bischof J. Hauser D. Schattel V. Hirner H.. (2009). 3,4-Diaryl-isoxazoles and -imidazoles as potent dual inhibitors of p38alpha mitogen activated protein kinase and casein kinase 1delta. J. Med. Chem. 52, 7618–7630. 10.1021/jm9005127 19591487

[B48] Perez D. I. Gil C. Martinez A. (2010). Protein kinases CK1 and CK2 as new targets for neurodegenerative diseases. Med. Res. Rev. 31, 924–954. 10.1002/med.20207 20577972

[B49] Poppema S. De Jong B. Atmosoerodjo J. Idenburg V. Visser L. De Ley L. (1985). Morphologic, immunologic, enzymehistochemical and chromosomal analysis of a cell line derived from Hodgkin's disease. Evidence for a B-cell origin of Sternberg-Reed cells. Cancer 55, 683–690. 3881158 10.1002/1097-0142(19850215)55:4<683::aid-cncr2820550402>3.0.co;2-o

[B50] Price M. A. (2006). CKI, there's more than one: casein kinase I family members in Wnt and Hedgehog signaling. Genes Dev. 20, 399–410. 10.1101/gad.1394306 16481469

[B51] Pulvertaft J. V. (1964). Cytology of Burkitt's Tumour (African Lymphoma). Lancet 1, 238–240. 14086209 10.1016/s0140-6736(64)92345-1

[B52] Pulvertaft J. V. (1965). A study of malignant tumours in Nigeria by short-term tissue culture. J. Clin. Pathol. 18, 261–273. 14304234 10.1136/jcp.18.3.261 PMC472922

[B53] Reed J. C. (2008). Bcl-2-family proteins and hematologic malignancies: history and future prospects. Blood 111, 3322–3330. 10.1182/blood-2007-09-078162 18362212 PMC2275002

[B54] Richter J. Bischof J. Zaja M. Kohlhof H. Othersen O. Vitt D.. (2014a). Difluoro-dioxolo-benzoimidazol-benzamides as potent inhibitors of CK1delta and epsilon with Nanomolar inhibitory activity on cancer cell proliferation. J. Med. Chem. 57, 7933–7946. 10.1021/jm500600b 25191940

[B55a] Richter J. Ullah K. Xu P. Alscher V. Blatz A. Peifer C.. (2014b). Effects of altered expression and activity levels of CK1δ and ε on tumor growth and survival of colorectal cancer patients. Int. J. Cancer. [Epub ahead of print]. 10.1002/ijc.29346 25404202

[B55] Rivers A. Gietzen K. F. Vielhaber E. Virshup D. M. (1998). Regulation of casein kinase I epsilon and casein kinase I delta by an *in vivo* futile phosphorylation cycle. J. Biol. Chem. 273, 15980–15984. 9632646 10.1074/jbc.273.26.15980

[B56] Schittek B. Sinnberg T. (2014). Biological functions of casein kinase 1 isoforms and putative roles in tumorigenesis. Mol. Cancer 13, 231. 10.1186/1476-4598-13-231 25306547 PMC4201705

[B57] Schmitz R. Stanelle J. Hansmann M. L. Kuppers R. (2009). Pathogenesis of classical and lymphocyte-predominant Hodgkin lymphoma. Annu. Rev. Pathol. 4, 151–174. 10.1146/annurev.pathol.4.110807.092209 19400691

[B58] Schneider R. K. Adema V. Heckl D. Jaras M. Mallo M. Lord A. M.. (2014). Role of casein kinase 1A1 in the biology and targeted therapy of del(5q) MDS. Cancer Cell 26, 509–520. 10.1016/j.ccr.2014.08.001 25242043 PMC4199102

[B59] Schneider U. Schwenk H. U. Bornkamm G. (1977). Characterization of EBV-genome negative “null” and “T” cell lines derived from children with acute lymphoblastic leukemia and leukemic transformed non-Hodgkin lymphoma. Int. J. Cancer 19, 621–626. 68013 10.1002/ijc.2910190505

[B60] Stein H. Warnke R. A. Chan W. C. Jaffe E. S. Chan J. K. C. Gatter K. C. (2008). Diffuse large B-cell lymphoma, not otherwise specified, in WHO Classification of Tumours of Haematopoietic and Lymphoid Tissues, 4th Edn., eds Swerdlow S. H. Campo E. Harris N. L. Jaffe E. S. Pileri S. A. Stein H. Thiele J. Vardiman J. W. (Lyon: IARC), 233–237.

[B61] Stoter M. Bamberger A. M. Aslan B. Kurth M. Speidel D. Loning T.. (2005). Inhibition of casein kinase I delta alters mitotic spindle formation and induces apoptosis in trophoblast cells. Oncogene 24, 7964–7975. 10.1038/sj.onc.1208941 16027726

[B62] Stoter M. Kruger M. Banting G. Henne-Bruns D. Knippschild U. (2014). Microtubules depolymerization caused by the CK1 inhibitor IC261 may be not mediated by CK1 blockage. PLoS ONE 9:e100090. 10.1371/journal.pone.0100090 24937750 PMC4061085

[B63] Tsai I. C. Woolf M. Neklason D. W. Branford W. W. Yost H. J. Burt R. W.. (2007). Disease-associated casein kinase I delta mutation may promote adenomatous polyps formation via a Wnt/beta-catenin independent mechanism. Int. J. Cancer 120, 1005–1012. 10.1002/ijc.22368 17131344

[B64] Tsujimoto Y. Finger L. R. Yunis J. Nowell P. C. Croce C. M. (1984). Cloning of the chromosome breakpoint of neoplastic B cells with the t(14;18) chromosome translocation. Science 226, 1097–1099. 6093263 10.1126/science.6093263

[B65] Wang F. Liu J. Robbins D. Morris K. Sit A. Liu Y. Y.. (2011). Mutant p53 exhibits trivial effects on mitochondrial functions which can be reactivated by ellipticine in lymphoma cells. Apoptosis 16, 301–310. 10.1007/s10495-010-0559-8 21107702 PMC3078632

[B66] Yamane A. Resch W. Kuo N. Kuchen S. Li Z. Sun H. W.. (2011). Deep-sequencing identification of the genomic targets of the cytidine deaminase AID and its cofactor RPA in B lymphocytes. Nat. Immunol. 12, 62–69. 10.1038/ni.1964 21113164 PMC3005028

